# Knowledge and awareness toward human papillomavirus vaccination among Saudi female nursing students

**DOI:** 10.3389/fgwh.2024.1470048

**Published:** 2024-10-24

**Authors:** Amal Khulaif Alanazi, Eithar Kayal, Shahad Alanzi, Hanadi Al Hodian, Alhanouf Bin Rusayes

**Affiliations:** ^1^ Nursing Department, College of Nursing, King Saud Bin Abdulaziz University for Health Sciences, Riyadh, Saudi Arabia; ^2^ King Abdullah International Medical Research Center, Riyadh, Saudi Arabia

**Keywords:** human papillomavirus, knowledge, nursing, vaccine, cervical cancer

## Abstract

**Introduction:**

Human Papillomavirus is a sexually transmitted agent, causing cervical cancer. In Saudi Arabia, cervical cancer is ranked as the ninth most common carcinoma in women. HPV vaccine is an effective prevention method against HPV high-risk types such as HPV 18 and 16. Research on HPV vaccine knowledge and awareness is limited due to the lack of extensive data reportage on HPV and cervical cancer cases among Saudi women.

**Aim:**

This study was aimed to determine the knowledge and awareness of human papillomavirus vaccination among Saudi nursing female students.

**Methods:**

This study was cross-sectional and included (*n* = 114) participants. The study used an online survey, which included demographical variables and the HPV knowledge scale. The data were collected from October 10, 2023, until January 3, 2024. Descriptive data, Mann-Whitney Z-tests and nonparametric tests were used to analyze the study's findings.

**Results:**

The study participants’ mean age was 20.8 years (SD 1.6). Most students (72%) did not receive the HPV vaccine. The overall mean HPV knowledge was 10.0 (SD 7.08). The HPV knowledge subscales showed poor levels of knowledge of HPV infection, screening, and vaccines: 5.15 (SD 3.87), 1.39 (SD 1.34), and 2.06 (SD 1.87), respectively.

**Discussion:**

In conclusion, Saudi Arabia having a predominantly youthful population, it is crucial to implement educational programs that improve the understanding and awareness of HPV infection among nursing students and other health professionals. There is a necessity to establish impactful awareness campaigns and integrate interventional research to inform health professionals and the public about the disease and dispel misunderstandings and cultural beliefs about HPV and HPV vaccines to prevent cervical cancers among young females.

## 1 Introduction

Human Papillomavirus (HPV) is a virus that can cause abnormal tissue growth, such as warts and other cell changes, leading to the development of many malignancies, including cervical cancer. Current research findings indicate that HPV infection is the most common sexually transmitted infection worldwide, with an incidence of 11.7% (1). In Saudi Arabia, the incidence and prevalence rate of HPV is underrepresented due to the lack of extensive data reportage on HPV and cervical cancer screening programs (2, 3). This underscores the urgent need for more comprehensive data on HPV in Saudi Arabia. However, some studies were conducted and showed an inconsistent prevalence rate of HPV that ranged from 2.2% to 43% among Saudi women (4–7).

Human papillomavirus is considered the main etiological factor of cervical cancer which is the fourth most common malignancy in women globally (1). HPV viruses are divided into low-risk and high-risk categories based on their oncological pathogenesis. The two low-risk HPV types that are most frequently found, HPV 6 and 11, which are responsible for genital warts, whereas the two high-risk HPV types, HPV 16 and 18, are responsible for cancer, notably cervical, oropharyngeal, anal, vaginal, vulvar, and penile malignancies (8, 9). Cervical cancer is ranked as the ninth most common carcinoma in Saudi women (10). Recent reports indicate that 10.7 million Saudi women aged 15 and older are at risk of developing cervical cancer. Current estimates show that 358 women are diagnosed with cervical cancer and 179 die from it yearly (11).

Human papillomavirus vaccines are an essential and cost-effective prevention method against HPV infection and prevent more than 90% HPV related malignancies specifically cervical cancer (8, 12). Since its utilization, the HPV vaccination has shown a reduction rate of 88% in adolescent girls and women and 81% in men for HPV-related cancers and genital warts (13, 14). There are three HPV vaccines available, the HPV 9-valent vaccine (Gardasil 9, 9vHPV), the HPV quadrivalent vaccine (Gardasil, 4vHPV), and the HPV bivalent vaccine (Cervarix, 2vHPV) has been approved by the United States Food and Drug Administration (13, 14). The currently utilized HPV vaccine in the United States is the Gradisl-9, and CDC approves vaccines as a series of 2 or 3 doses. A two-dose series (0, 6–12 months) for most individuals who initiate vaccination at ages 9 through 14 years, and A three-dose series (0, 1–2, 6 months) for individuals who start vaccination at ages 15 through 45 years and for immunocompromised individuals (13–15). The Saudi Food and Drug Authority (SFDA) approved the HPV vaccine for females ages 11 to 26 in 2010 (16). The immunizations are recommended for school age children and young adults from 9 up to 26 years by the Saudi Ministry of Health and currently accessible in Saudi Arabia's major hospitals and at public school settings (17, 18).

### 1.1 Knowledge of human papillomavirus

In the literature, many studies were conducted and most of these studies showed low knowledge levels and poor awareness of human papillomavirus in middle east and in Saudi Arabia (2, 19, 20). In a study in Jordan, the level of knowledge of HPV was low, and the decrease in knowledge was higher among nursing students, as only one-third of the participants were aware of HPV (20). In addition, a review of studies on human papillomavirus demonstrated that 84.06% on a sample of Saudi women did not know any information of HPV (5). Also, recent evidence mentioned a decrease in the number of public health programs targeting awareness of the HPV infection and the procedures associated with its prevention (21). In another study, 15% of participants had prior awareness of HPV infection, while 34.5% had misconceptions and poor awareness about HPV (22). These findings indicate the need for more research on assessment of human papillomavirus knowledge and awareness levels specifically among nursing students as they are well positioned to raise awareness of HPV infection and its prevention methods.

### 1.2 Awareness of human papillomavirus

Awareness of the human papillomavirus virus and its vaccine is crucial in preventing cervical cancer development among females. A recent study by Al Mehmadi (22) revealed poor awareness levels among a sample of 1,033 males and females, with only 25.9% demonstrating good knowledge of HPV (22). Aldawood et al. (2) further investigated awareness among college students and found that 40% of the study sample had never heard of HPV, with noticeable differences in the level of awareness among male and female students. Their study also indicated that increased age and type of health profession, such as medicine, led to higher awareness among students (2). These findings underscore the existing gaps in awareness of HPV and its vaccine among college students, highlighting the need to explore personal factors such as level of education and age that may influence the level of awareness of HPV among students. This research has the potential to significantly impact public health by addressing these awareness gaps**.**

### 1.3 Knowledge of human papillomavirus vaccine

In 2010 the Kingdom of Saudi Arabia approved the availability of preventative HPV vaccines, including the bivalent vaccine Cervarix and the quadrivalent vaccine Gardasil, for girls between the ages of 11 and 26 (16). In a study conducted on Saudi women, 1.9% of the study's participants took the vaccine (10). Similarly, a study was conducted on a sample of 645 individuals in the eastern region showed that only 4% of the sample have received their HPV vaccination (23). The reasons for not taking vaccine were lack of HPV vaccine information, risks associated with the vaccine and participants believed that they are healthy and no need to be vaccinated (23).

However, recent studies demonstrated that 78% of HPV high risk genotypes such as 16 and 18 were present in 92% of cervical tumors and most of the women in these studies were diagnosed at advanced stages of cervical cancer due to lack of regular cytology screening (24, 25). The Saudi Ministry of Health (MOH) published HPV screening practice guidelines in 2014, but due to the reported low incidence of cervical cancer and from the standpoint of cost effectiveness, there isn't yet a national screening program for HPV and cervical cancer in Saudi Arabia; however, in 2022 the Saudi MOH included the HPV vaccine in the national immunization schedule (25–27). Therefore, studies targeting HPV vaccination is still warranted to raise awareness of receiving the vaccination to protect women against many malignancies including cervical cancer. These studies suggest that more research is needed to address the gaps relating to HPV knowledge, and awareness among Saudi females. Therefore, this study was aimed to determine the knowledge and awareness of human papillomavirus vaccination among Saudi nursing female students. Additionally, this study examined the student's source of information on HPV infection and its vaccine to gain insights and identify effective strategies to prevent the development of HPV-related diseases among this population. This study's purpose aligned with the Saudi vision for healthcare 2030 (28) to enhance preventive care for young women and optimize their health.

## 2 Materials and methods

### 2.1 Study design, and setting

This study was a cross-sectional design. The study was conducted at King Saud University for Health Sciences (KSAU-HS) at colleges of nursing in three campuses Riyadh, Al Ahsa, and Jeddah. King Saud bin Abdulaziz University for Health Sciences (KSAU) is a governmental university specializing in health sciences.

### 2.2 Sample size and sampling

The study focused on nursing students enrolled in nursing program at KSAU university in year 2023–2024. The sample was calculated using the Raosoft software (Sample Size Calculator; Raosoft Inc.) with margin error of 5%, confidence interval of 95% total population of 891, the recommended sample was 269 (29).The current study had a total of 114 and response rate was 42.4%. The sample Inclusion criteria were as follows: (1) all female nursing students enrolled in King Saud University for Health Sciences in the 2023–2024 academic year (2) age between 17 and 26 which is the average age for college students and fall within the age group of receiving the vaccine (3) Saudi nationality (4) able to read and speak English Language.

### 2.3 Data collection and ethical consideration

The data were collected from October 10, 2023, until January 3, 2024. Quantitative data was gathered using a web survey and the researchers sent an email invitation to nursing students in three university regions. The study's team sent detailed information about the study's objective, risks, and benefits. Interested participants were instructed to fill out an informed consent and complete the link to the study's questionnaires. This study's ethic has been approved by King Abdullah International Medical Research Center, Institutional Review Board, SP23R-157-06, IRB#1737/23.

### 2.4 Measures

Data was measured using a structured online survey of 34 item questionnaire, excluding demographical variables. The first section included questions about demographical data such as age, education level, marital status, region of residence, the status of HPV vaccine, mother's education level, and smoking or e-cigarettes (vaping) use. Questions regarding knowledge and awareness of HPV infection, vaccination, and testing was measured using an existing validated published instrument (30). Questions concerning vaccine and HPV testing was obtained from Centers for Disease Control and Prevention and the Saudi Ministry of Health (31).

The knowledge of students regarding HPV screening, vaccine, and vaccine availability has been assessed by using a 34-item questionnaire, where the “true” coded with 1 “false” and “I don't know” coded with 0 as the answer options. Negative questions have been re-coded inversely to avoid bias in the score. The total knowledge score has been calculated by adding all 34 items, and a score range from 0 to 34 has been generated; the higher the score, the higher the knowledge of HPV screening, vaccine, and vaccine availability. By using 50% and 75% as cutoff points to determine the level of knowledge, students were considered as having poor knowledge if the score was below 50%, 50% to 75% were considered moderate, and above 75% were considered as having a good knowledge level. The general awareness about HPV was assessed using a 3-item questionnaire: HPV infection, HPV testing, and HPV vaccination. Respondents were considered to have good awareness if they had heard at least 2 HPV questions, while respondents who had heard one or had not heard any of the HPV questions were considered to have poor knowledge. The reliability test of the 34-item questionnaire has a Cronbach Alpha of 0.952 or 95.2%, indicating an excellent internal consistency of this study questionnaire.

#### 2.4.1 The HPV knowledge scale

This scale has 28 items with subscales of knowledge, vaccine, and testing regarding HPV infection. We modified one statement after obtaining approval from the authors of the instrument to maintain cultural sensitivity (30). In the second section of the survey, we assessed the HPV knowledge with a 15-item questionnaire. Participants was first be asked, “Before today, had you ever heard of human papillomavirus?” Those who answered “yes” were asked to complete the HPV knowledge scale in a “True/False/don't know” format. In this section, participants were asked about types and patterns of transmission, risk factors, preventive measures, signs, symptoms, treatment, and cervical cancer. Each correct response was given a score of 1, and each incorrect or “I Don't Know” response was given a score of 0. As such, the general HPV knowledge score could range from 0 to 15 and higher scores indicate a greater HPV knowledge.

The knowledge of the HPV testing subscale was assessed in the third section of study's survey with a 6-item questionnaire. Participants were first asked, “Have you ever heard of HPV testing?” Those who answered “yes” were asked to complete the knowledge of the HPV testing scale in a “True/False/Don't know” format. This scale included information about the knowledge of the Pap test and HPV test, a positive test for HPV, and its relation to cervical cancer. Each correct response was given a score of 1, and each incorrect or “I Don't Know” response was given a score of 0. As such, the knowledge of HPV testing scores could range from 0 to 7, with higher scores indicating greater knowledge of HPV testing.

The HPV vaccine knowledge subscale was assessed in the fourth section with a 7-item questionnaire. Participants were first asked, “Before today, had you ever heard of HPV vaccination?” Those who answered “yes” were asked to complete the HPV Vaccine Knowledge Scale in a “True/False/Don't know” format. The next items assessed their knowledge of the dose, the need for Pap smear testing among HPV-vaccinated individuals, and the protective role of the vaccine against sexually transmitted diseases, genital warts, and cervical cancer. Each correct response was given a score of 1, and each incorrect or “I Don't Know” response was given a score of 0. As such, the HPV Vaccine Knowledge scores could range from 0 to 7, with higher scores indicating greater knowledge about the HPV Vaccine.

The fourth and final section addressed descriptive data on the availability of the HPV vaccine in the Kingdom of Saudi Arabia. The 6 items were “True/False/I Don't Know” questions, which asked about the recommended age for taking the vaccine, the authorization of the papillomavirus vaccine for women, the protective role of the vaccine against genital warts and cervical cancer, and the recommendation for the male HPV vaccine. Each correct response was given a score of 1, and each incorrect or “I Don't Know” response was given a score of 0. As such, the availability of HPV vaccination in the KSA scores could range from 0 to 7. The total scale score was calculated as the sum of all subscales for a total knowledge score of 28 items, excluding the calculation of demographic data. Cronbach's alpha for the original scale was 0.849 (30).

### 2.5 Statistical analysis

The data were analyzed using the software program Statistical Packages for Software Sciences (SPSS) version 26 (Armonk, New York, IBM Corporation, USA). For the descriptive analysis, the mean ± SD was used for metric variables while number and percentage (%) were given for categorical variables. The differences in the knowledge score according to socio-demographic characteristics were carried out using the Mann-Whitney Z-test. The normality test was performed using the Shapiro-Wilk test as well as Kolmogorov-Smirnov Test. The knowledge score follows the non-normal distribution. Thus, the non-parametric tests were applied. Furthermore, the relationship between the level of awareness and the students’ socio-demographic characteristics has been conducted using the Chi-square test. Values were considered significant with a *p*-value of less than 0.05.

## 3 Results

### 3.1 Sample characteristics

This study enrolled 114 Saudi nursing students with a response rate of 42.4% as seen in Table 1, 60.5% were aged between 21 and 25 years old. Most of the students were single (95.6%). Students who were in year level 4 constituted 36%. The majority (60.5%) were studying at Riyadh. The prevalence of students who received HPV vaccination was 28.1%, while the prevalence of smokers was 9.6%. Regarding mother's education, 43.9% had bachelor's or higher degrees. In addition, 59.6%, 30.7%, and 30.7% have heard of HPV, HPV testing and HPV vaccination.

**Table 1:** Socio-demographic characteristics of the Saudi female nursing students (*n* = 114).

Study variables	*N* (%)
Age group	
• 17–20 years	45 (39.5%)
• 21–25 years	69 (60.5%)
Marital status	
• Single	109 (95.6%)
• Married	05 (04.4%)
Academic year	
• Year 1	17 (14.9%)
• Year 2	12 (10.5%)
• Year 3	25 (21.9%)
• Year 4	41 (36.0%)
• Year 5	16 (14.0%)
• Year 6	03 (02.6%)
City of residence	
• Riyadh	69 (60.5%)
• Al Ahsa	06 (05.3%)
• Jeddah	39 (34.2%)
Status of HPV vaccination	
• Yes - Received HPV vaccine	32 (28.1%)
• No - Did not receive HPV vaccine	82 (71.9%)
Smoking or e-cigarette (vaping) use	
• Yes	11 (09.6%)
• No	103 (90.4%)
Mother educational level	
• Primary School	19 (16.7%)
• Middle School	13 (11.4%)
• High School	32 (28.1%)
• Bachelor's Degree or higher	50 (43.9%)
Heard of HPV	
• Yes	68 (59.6%)
• No	34 (29.8%)
• I don't know	12 (10.5%)
Heard of HPV testing	
• Yes	35 (30.7%)
• No	65 (57.0%)
• I don't know	14 (12.3%)
Heard of HPV vaccination	
• Yes	35 (30.7%)
• No	65 (57.0%)
• I don't know	14 (12.3%)

### 3.2 The students’ knowledge of HPV infection, testing, vaccination and HPV vaccine availability

Regarding the knowledge of students about HPV (Table 2), the top three statements with the highest rating of correct answers were “HPV can be passed on during sexual intercourse” (56.1%), “HPV can cause cervical cancer” (46.5%), and “HPV can be passed on by genital skin-to-skin contact” (56.1%), while the statements such as “HPV usually doesn't need any treatment” (12.3%), “HPV can cause HIV/Aids” (19.3%), and “Having sex at an early age increases the risk of getting HPV” (21.1%) showed the least ratings. The total mean HPV knowledge score was 5.15 (SD 3.87).

**Table 2:** Assessment of knowledge toward HPV, its screening, vaccine and vaccine availability (*n* = 114).

Knowledge domains	*N* (%)
Knowledge about HPV score (mean ± SD)	5.15 ± 3.87
1. HPV can be passed on during sexual intercourse [true]	64 (56.1%)
2. HPV can cause cervical cancer [true]	53 (46.5%)
3. HPV can be passed on by genital skin-to-skin contact [true]	53 (46.5%)
4. Using condoms reduces the risk of getting HPV [true]	51 (44.7%)
5. HPV can cause genital warts [true]	46 (40.4%)
6. A person could have HPV for many years without knowing it [true]	43 (37.7%)
7. HPV is very rare [false]	42 (36.8%)
8. Men cannot get HPV [false]	39 (34.2%)
9. There are many types of HPV [true]	39 (34.2%)
10. Most sexually active people will get HPV at some point in their lives [true]	34 (29.8%)
11. HPV can be cured with antibiotics [false]	33 (28.9%)
12. HPV always has visible signs or symptoms [false]	30 (26.3%)
13. Having sex at an early age increases the risk of getting HPV [true]	24 (21.1%)
14. HPV can cause HIV/Aids [false]	22 (19.3%)
15. HPV usually doesn't need any treatment [true]	14 (12.3%)
Knowledge about HPV screening score (mean ± SD)	1.39 ± 1.34
16. If a woman tests positive for HPV she will definitely get cervical cancer [false]	41 (36.0%)
17. An HPV test can be done at the same time as a Pap test [true]	31 (27.2%)
18. If an HPV test shows that a woman does not have HPV, her risk of cervical cancer is low [true]	28 (24.6%)
19. HPV testing is used to indicate if the HPV vaccine is needed [true]	25 (21.9%)
20. An HPV test can tell you how long you have had an HPV Infection [true]	19 (16.7%)
21. When you have an HPV test, you get the results the same day [true]	14 (12.3%)
Knowledge about HPV vaccine score (mean ± SD)	2.06 ± 1.87
22. One of the HPV vaccines offers protection against genital warts [true]	43 (37.7%)
23. The HPV vaccines offer protection against all sexually transmitted infections [false]	43 (37.7%)
24. Girls who have had an HPV vaccine do not need a Pap test when they are older [false]	40 (35.1%)
25. Someone who has an HPV vaccine cannot develop cervical cancer [false]	38 (33.3%)
26. HPV vaccines offer protection against most cervical cancers [true]	30 (26.3%)
27. The HPV vaccines are most effective if given to people who have never had sex [true]	22 (19.3%)
28. The HPV vaccine requires three doses [true]	19 (16.7%)
Knowledge about HPV vaccine availability score (mean ± SD)	1.42 ± 1.50
29. HPV vaccine is recommended for all females ages 11–26 years [true]	49 (43.0%)
30. The National Immunization Schedule provides free HPV vaccines [true]	44 (38.6%)
31. HPV vaccine is permitted for males aged 11–26 years [true]	22 (19.3%)
32. The HPV vaccine is usually given to girls in school settings [false]	19 (16.7%)
33. HPV vaccine is licensed for women aged 30–45 years [false]	17 (14.9%)
34. HPV vaccines that are available and approved by Saudi Food and Drug Authority (Gardasil Recombinant vaccine) protect against both genital warts and cervical cancer [false]	11 (09.6%)
Total knowledge score (mean ± SD)	10.0 ± 7.08
Level of knowledge	
• Poor	93 (81.6%)
• Moderate	20 (17.5%)
• Good	01 (0.90%)

Regarding the knowledge about HPV screening, poor knowledge was seen in all statements, particularly in the statement related to “When you have an HPV test, you get the results the same day” (12.3%), “An HPV test can tell you how long you have had an HPV Infection” (16.7%), and “HPV vaccines offer protection against most cervical cancers” (21.9%). The total mean knowledge score of HPV screening was 1.39 (SD 1.34).

**Figure 1:**
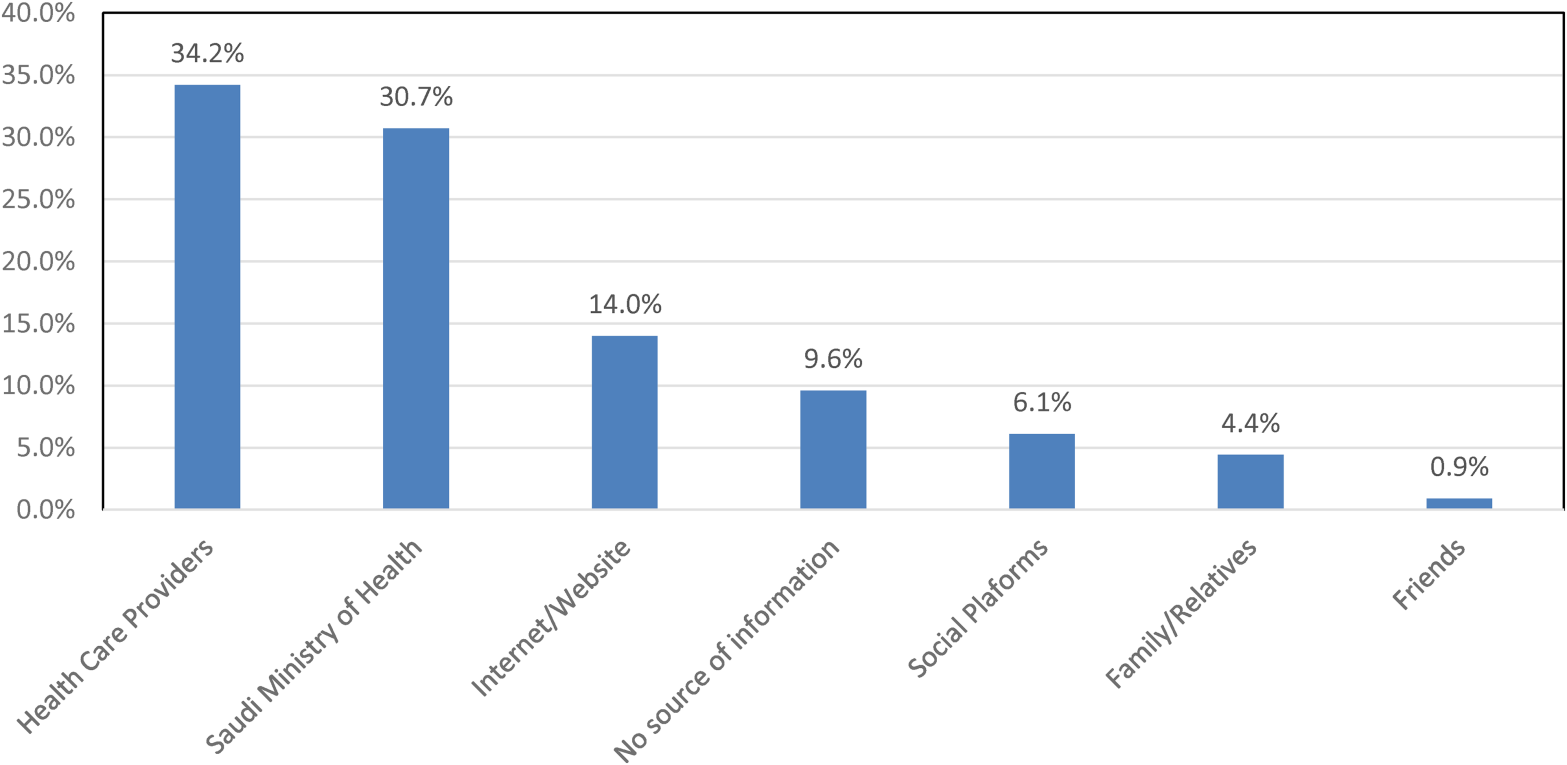
Sources of HPV information.

Regarding the knowledge about the HPV vaccine, unsatisfactory knowledge was seen in all statements, notably on “The HPV vaccine requires three doses” (16.7%), “The HPV vaccines are most effective if given to people who have never had sex” (19.3%) and “HPV vaccines offer protection against most cervical cancers” (26.3%). The total mean knowledge score of the HPV vaccine was 2.06 (SD 1.87). Also, this study showed that the most common source of HPV information was health care providers (34.2%), followed by Saudi Ministry of Health (30.7%) and Internet/Website (14%) as illustrated in Figure 1.

Finally, regarding the knowledge about vaccine availability, insufficiency of knowledge was seen in all statements, particularly about “HPV vaccines that are available and approved by Saudi Food and Drug Authority (Gardasil Recombinant vaccine) protect against both genital warts and cervical cancer” (9.6%), “HPV vaccine is licensed for women aged 30–45 years” (14.9%), and “The HPV vaccine is usually given to girls in school settings” (19.3%). The total mean knowledge of HPV vaccine availability was 1.42 (SD 1.50).

The overall mean score of the knowledge about HPV was 10.0 (SD 7.08), with poor, moderate, and good knowledge levels consisting of 81.6%, 17.5%, and 0.9%, respectively. When measuring the differences in the score of knowledge in relation to the socio-demographic characteristics and general awareness toward HPV (Table 3), it was observed that a higher knowledge score was more associated with being older (*Z* = 2.673; *p* = 0.008), being a senior student (*Z* = 3.338; *p* = 0.001), having received HPV vaccination (*Z* = 2.173; *p* = 0.030), having heard of HPV (*Z* = 5.180; *p* < 0.001), having heard of HPV testing (*Z* = 4.357; *p* < 0.001) and having heard of HPV vaccination (*Z* = 5.206; *p* < 0.001).

**Table 3:** Differences in the score of knowledge in relation to the socio-demographic characteristics and general awareness of HPV among the Saudi female nursing students (*n* = 114).

Factor	Knowledge score (34) Mean ± SD	*Z*-test	*P*-value*
Age group			
• 17–20 years	7.98 ± 8.26	2.673	0.008**
• 21–25 years	11.3 ± 5.88		
Academic year level			
• Junior students (Year 1–3)	7.83 ± 8.01	3.338	0.001**
• Senior students (Year 4–6)	11.9 ± 5.48		
City of residence			
• Inside Riyadh	10.3 ± 7.34	0.559	0.576
• Outside Riyadh	9.58 ± 6.71		
Status of HPV vaccination			
• Yes - Received HPV vaccine	12.4 ± 6.97	2.173	0.030**
• No - Did not receive HPV vaccine	9.09 ± 6.94		
Smoking or e-cigarette (vaping) use			
• Yes	12.8 ± 6.49	1.210	0.226
• No	9.72 ± 7.10		
Mother educational level			
• High School or below	10.0 ± 7.26	0.120	0.904
• Bachelor's Degree or higher	10.0 ± 6.91		
Heard of HPV^a^			
• Yes	12.9 ± 5.74	5.180	<0.001**
• No	5.32 ± 6.33		
Heard of HPV testing^a^			
• Yes	14.6 ± 6.05	4.357	<0.001**
• No	7.97 ± 6.56		
Heard of HPV vaccination^a^			
• Yes	12.8 ± 6.08	5.206	<0.001**
• No	5.41 ± 6.07		

* *P*-value has been calculated using Mann Whitney Z-test.

** Significant differences between groups at *p* < 0.05.

^a^ Students who said “I don't know” were excluded from the statistical test.

### 3.3 The students’ awareness of HPV infection, testing, vaccination and HPV vaccine availability

**Figure 2:**
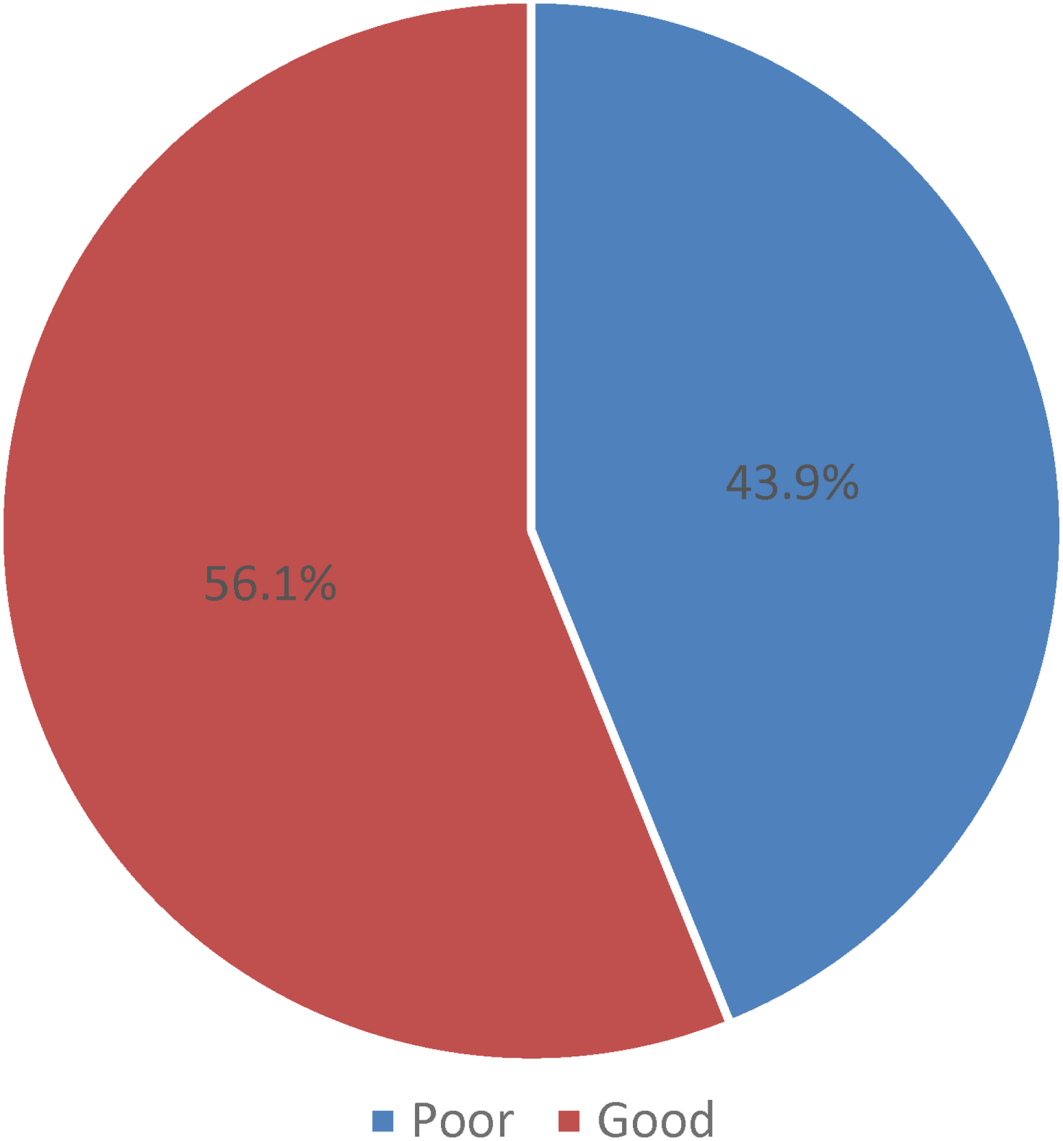
Level of awareness toward HPV.

**Figure 3:**
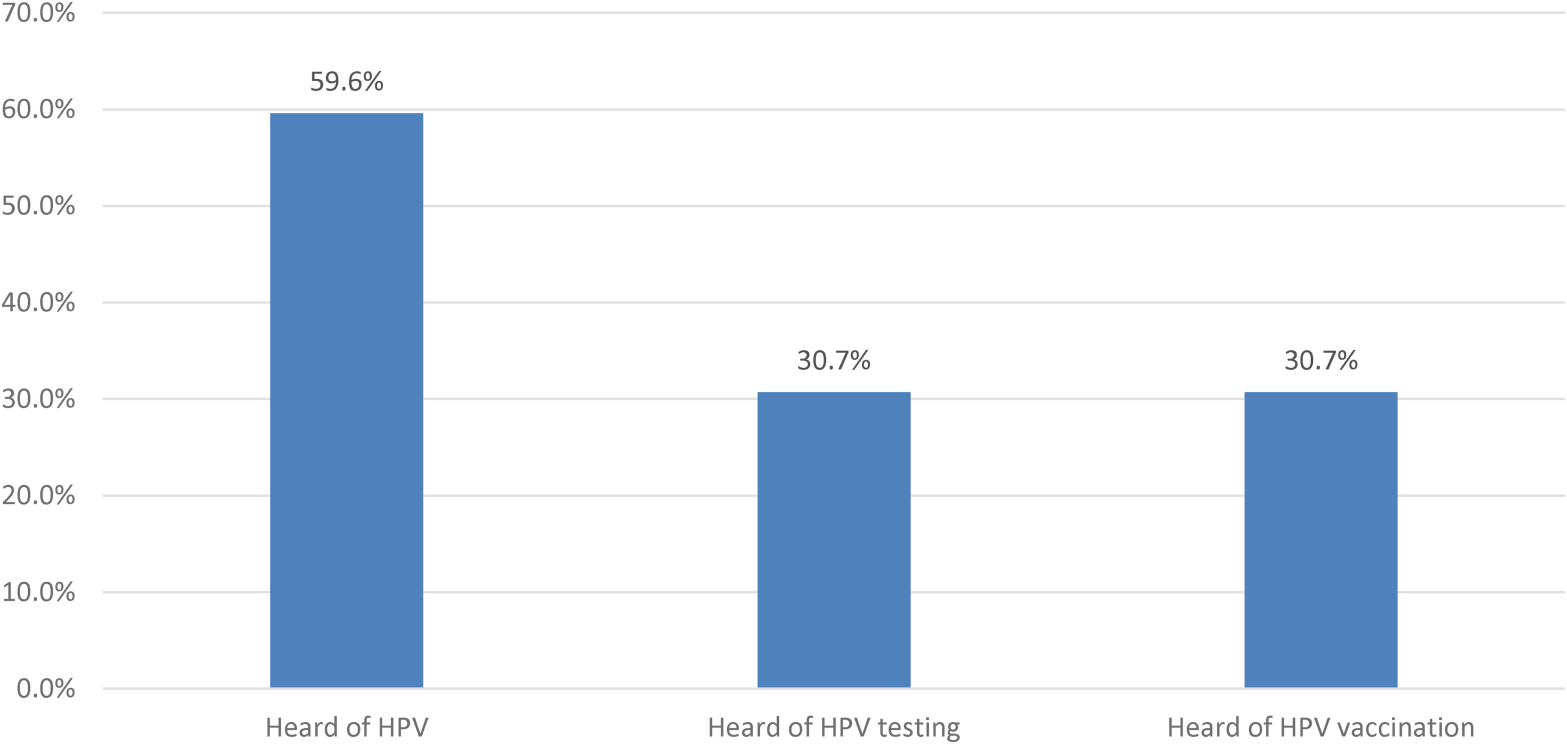
Assessment of the awareness about HPV infection, testing, and vaccination.

The students in this study had a 43.9% poor level of awareness about HPV while the rest were having good awareness levels of HPV (56.1%) as illustrated in Figure 2. Furthermore, the students showed that the awareness assessment about HPV infection, testing, and vaccination were observed as 59.6% have heard about HPV, and 30.7% have heard about HPV testing and vaccination as shown in Figure 3. Measuring the relationship between the level of awareness and the socio-demographic characteristics of the student nurses found that increasing age (*p* = 0.005) and increasing academic year level (*p* < 0.001) were associated with a good level of awareness. No significant relationships were observed between the level of awareness in relation to the city of residence, status of HPV vaccination, smoking, and mother education (*p* > 0.05) (Table 4).

**Table 4:** Relationship between the level of awareness and the socio-demographic characteristics and general awareness of HPV among Saudi female nursing students (*n* = 114).

Factor	Level of awareness		*P*-value*
	Poor *N* (%) ^(*n* = 50)^	Good *N* (%) ^(*n* = 64)^	
Age group			
• 17–20 years	27 (54.0%)	18 (28.1%)	0.005**
• 21–25 years	23 (46.0%)	46 (71.9%)	
Academic year level			
• Junior students (Year 1–3)	33 (66.0%)	21 (32.8%)	<0.001**
• Senior students (Year 4–6)	17 (34.0%)	43 (67.2%)	
City of residence			
• Inside Riyadh	31 (62.0%)	38 (59.4%)	0.776
• Outside Riyadh	19 (38.0%)	26 (40.6%)	
Status of HPV vaccination			
• Yes - Received HPV vaccine	10 (20.0%)	22 (34.4%)	0.090
• No - Did not receive HPV vaccine	40 (80.0%)	42 (65.6%)	
Smoking or e-cigarette (vaping) use			
• Yes	04 (08.0%)	07 (10.9%)	0.598
• No	46 (92.0%)	57 (89.1%)	
Mother educational level			
• High School or below	29 (58.0%)	35 (54.7%)	0.727
• Bachelor's Degree or higher	21 (42.0%)	29 (45.3%)	

* *P*-value has been calculated using Chi-square test.

** Significant differences between groups at *p* < 0.05.

## 4 Discussion

This study examined the knowledge and awareness of human papillomavirus infection among Saudi nursing students. The findings demonstrated poor knowledge across all domains of the HPV knowledge scale. Specifically, participants showed a poor overall knowledge score, accounting for 81.6%. Knowledge of HPV had the highest mean compared to subscales of HPV screening, vaccine, and vaccine availability. Similarly, Darraj et al. (3) conducted a study among 569 adults residing in the Jazan Province, southeast of Saudi, with only 20% indicating overall good knowledge of HPV.

Furthermore, the results showed that nearly half of the students identified HPV as a sexually transmitted disease, and 46.3% believed it can cause cervical cancer. The students’ HPV knowledge scores were on the lowest side for information about HPV transmission, symptoms of the disease, and prevention methods such as using barrier contraception. The students had misconceptions about HPV treatments, with the lowest score on the antibiotic statement. These findings align with another research by Farsi et al. (19), who reported that 517 male medical students had poor knowledge of HPV and HPV vaccines, and 73.7% of the sample had heard about the vaccine but with inaccurate information about the disease and its transmission method. This indicates that majority of young women lack knowledge on HPV infection and highlights the need to raise awareness about HPV and its prevention modalities among youth population.

Moreover, HPV screening and vaccine knowledge of students were low, as 36% new that having a positive HPV test did not necessarily result in cervical cancer and only 27.2% understands HPV/pap smear co testing for prevention of cervical cancer. In addition, students lacked information on screening statements about how HPV is risk factor for cervical cancer, when they should receive the vaccine and number of doses required for prevention. Consistently, A qualitive study by Jradi & Bawazir (32) interviewed 77 Saudi women of reproductive age reported that most women had poor knowledge about the screening especially if they had no signs of HPV infection. With the lowest gaps relating to screenings of HPV and cervical cancer, it is essential to prioritize knowledge on HPV vaccine and early screenings benefits.

This study showed 43.9% poor overall awareness of HPV among female nursing students. Regarding the HPV and HPV vaccine awareness, the study indicated that 68 students have heard of HPV infection, and 35 students heard about HPV testing and vaccines. These findings are in line with recent research on HPV and HPV vaccine awareness among college students (33, 34). In their study, Ogbolu and Kozlovszky (33) reported a relatively similar awareness level of 34.8% of HPV infection, and only 25% were about the vaccine. This lack of awareness could be related to the limited availability of resources on HPV infection and its vaccine. In the kingdom, the Ministry of Health included the HPV vaccine in 2022 in the national immunization schedule (26). It is relatively new and might explain why many students might not have heard about the vaccine and its benefits. Thus, it is essential to increase knowledge and awareness regarding HPV infection and HPV vaccine among young women through interventional research, educational programs, and campaigns that aim to enhance vaccination rates and other cervical cancer preventive measures.

This study also examined the role of specific demographics including age and education level in relation to knowledge and awareness scores. The findings demonstrated that nursing students aged 21–25 had higher scores compared to younger students which could be because of seniority in the field and better awareness of HPV infection and vaccination. Also, the results of the study showed that senior nursing students who received vaccination had a higher knowledge level. These findings is in line with recent research that found that higher academic level of a student was associated with the high knowledge score (35). In addition, Aneela et al., reported higher educational background of (43.1%) increased the knowledge of HPV compared to less than high school education (24.5%). All of which demonstrates consistency in how education can have a positive impact on the over-all level of knowledge. In addition, the results showed that the mother's educational level was not associated with higher knowledge score of the students, this possibly related to small sample size. However, other studies have shown that mother and parents’ education was positively related to the knowledge of their children on HPV (26). Other demographical data such region of residence and smoking or vaping was not associative with lower knowledge score, and it might be related to relatively lower rates of students in other campuses compared to Riyadh.

The study identified that “Health Care Provider” was the most common source of information (34.2%), possibly due to their involvement in the health care field. Furthermore, Salima et al. reported similar results in a study among college students in South Carolina, which showed 68% of students had a “Health Care Provider” as a source of knowledge. On the contrary, Aga et al. (35) conducted a similar study which showed the most common source of knowledge regarding HPV among nursing students was “Self-learning” (28.3%) while “Faculty”, “Hospital”, and “Curriculum” demonstrated 2.0%, 5.1%, and 13.1%, respectively. Therefore, identifying the students’ sources of information would assist in future planning and designing of educational programs targeting HPV and its vaccine from reliable and validated sources.

### 4.1 Limitations

This study showed valuable information about the level of knowledge and awareness among nursing students. However, there are some limitations, the small sample size and population in this study were nursing students of the Jeddah, Riyadh, and Al Ahsa campuses of King Saud bin Abdulaziz University for Health Sciences, Saudi Arabia, and, consequently, the results do not necessarily reflect that of the general population. Also, this study was cross-sectional and might limit our understanding on changes of knowledge levels over time. Furthermore, the online self-administered questionnaire for data collection carried a risk of recall bias or contamination from the students who participated. Another limitation was related to gender because the sample relied mainly on female students, thus, the knowledge of HPV and awareness among males were not examined due to the study setting. Further research is needed to address this gap in awareness and knowledge of HPV as it affects both males and females.

## 5 Conclusion

The study highlighted gaps in knowledge and awareness levels among nursing students regarding HPV infection, screenings, and HPV vaccine. The rising incidence of HPV infection and decreased vaccination rates is a worrisome issue. With Saudi Arabia having a predominantly youthful population, it is crucial to implement educational programs that improve the understanding and awareness of HPV infection among nursing students and other health professionals. Given the critical role of nursing students, increasing their awareness about HPV and its vaccine may contribute to promoting vaccination rates among the general population, who may lack knowledge about existing vaccines or be hesitant due to misconceptions in the community. Lastly, there is a necessity to establish impactful awareness campaigns and integrate interventional research to inform health professionals and the public about the disease and dispel misunderstandings and cultural beliefs about HPV and HPV vaccines to prevent cervical cancers among young females.

## Data Availability

The raw data supporting the conclusions of this article will be made available by the authors, without undue reservation.
